# Detection of *Candida albicans* Using a Manufactured Electrochemical Sensor

**DOI:** 10.3390/mi12020166

**Published:** 2021-02-08

**Authors:** Prakhar Dutta, Yi-Jung Lu, Han-Yu Hsieh, Tyng-Yuh Lee, Yi-Tzu Lee, Chao-Min Cheng, Yu-Jui Fan

**Affiliations:** 1 International Ph.D. Program for Biomedical Engineering, Graduate Institute of Biomedical Materials & Tissue Engineering, School of Biomedical Engineering, College of Biomedical Engineering, Taipei Medical University, 250 Wuxing St., Taipei 11031, Taiwan; emailprakhardutta@gmail.com; 2 Division of Family and Operative Dentistry, Department of Dentistry, Taipei Medical University Hospital, Taipei 11031, Taiwan; 893031@h.tmu.edu.tw; 3 Department of Signal Transduction, Research Institute for Microbial Disease, Osaka University, 3-1 Yamada-oka, Suita, Osaka 565-0871, Japan; h-yun@biken.osaka-u.ac.jp; 4 Institute of Biomedical Engineering, National Tsing Hua University, No. 101, Section 2, Kuang-Fu Road, Hsinchu 30013, Taiwan; leetyngyuh@gmail.com (T.-Y.L.); chaomin@mx.nthu.edu.tw (C.-M.C.); 5 Department of Emergency Medicine, Taipei Veterans General Hospital, Taipei 11217, Taiwan; s851009@yahoo.com.tw; 6 Faculty of Medicine, School of Medicine, National Yang-Ming University, Taipei 11221, Taiwan

**Keywords:** *Candida albicans*, electrochemical sensors, yeast cell

## Abstract

*Candida albicans* is a commensal fungus that is responsible for a lot of nosocomial infections in immunocompromised people. Cell culture is currently the predominant method for diagnosing candidiasis, but it is time consuming. In this study, we developed a rapid screen procedure by devising a method for detecting *C. albicans* with the use of electrochemical sensors. Through this experiment, we propose a method for the detection of *C. albicans* in the system through the use of personal glucose meters. The hemicellulase was used to break down the cell wall of *C. albicans* to glucose and oligo, which can be detected by a glucose meter. The spiked samples were prepared suspending *C. albicans* in urine and serum, demonstrating the feasibility of the developed method in a real situation.

## 1. Introduction

Candida are a species of fungi that can showcase polymorphism. Their ability to showcase polymorphism is one of the major factors for their pathogenesis in human hosts. *Candida albicans* is one of the most common yeasts found in the human body, and usually resides without any negative effects on the skin, gastrointestinal tract, urogenital tract, or oral cavity [1]. Blastoconidia or the yeast form can usually be related to asymptomatic colonization, as well as with the transmission or spread in the bloodstream [2]. In contrast, the mycelial or hyphal form contributes mostly to mucosal invasion and adherence [3], which are characteristics of disease with symptoms. Candida infections can be present in various parts of the body, and usually develop on mucous membranes such as the mouth or genitals, but the bloodstream can also be affected [4,5]. Invasive candidiasis (IC) is a serious infection caused by different species of candida that can affect the blood, heart, brain, eyes, bones, and other parts of the body. Usually, invasive candidiasis is associated with the penetration of *Candida* spp. into the tissue beneath the epithelium or an infection of the viscera [6]. Typical IC includes candidemia, chronic disseminated candidiasis, and dep-seated infection, where even if patients receive antifungal therapy, the mortality is as high as 40% for patients with invasive candidiasis [7]. Candidemia, the fourth most common bloodstream infection, is the most common type of IC in hospitalized patients [8]. An increase in incidence rates has been reported in most regions [9,10,11], and the maximum incidence rate of Candidemia has been observed at extreme ages [9,12,13]. The pathogenesis of invasive Candida can be concluded in several ways [14]. Candida species that colonize the gut invade through translocation or anastomotic leakage after laparotomy, and cause either B localized, deep-seated infection (e.g., peritonitis), or Candidemia. Women are highly susceptible to candida infections, and it has been seen that almost 75% of women suffer from vulvovaginal candidiasis (VVC) and 40–50% of women experience at least one or more episodes of infection [5,15]. It has recently been noted that there is an association between the genes that control *Candida albicans* morphogenesis and the immunopathology associated with VVC. Usually, healthcare providers and physicians use additional information such as medical history, physical exams, and symptoms, alongside laboratory testing, to diagnose invasive candidiasis. The current gold standard and the most widely used way for healthcare providers to test for invasive candidiasis involves taking a blood sample or a sample from the infected region and testing it in a laboratory to analyze if it will grow Candida in a culture [16]. However, usually, blood cultures take longer times to develop and are limited in their diagnostic ability because of poor sensitivity. As a result of the persisting problems with the current testing methods, new methods that either replace or complement the existing diagnostic methods are required in order to increase the diagnostic efficiency in terms of both the time required for diagnosis and the accuracy of diagnosis; in particular, to correctly diagnose the “missing 50%” of patients who show negative results in blood cultures. Using other methods of testing involving mannan/anti-mannan immunoglobulin G, polymerase chain reaction (PCR), and β-D-glucan (BDG) assays, it is possible to diagnose Candidemia before blood cultures, and these methods have shown promising sensitivity/specificity. Still, these methods are not widely investigated in blood culture-negative, deep-seated candidiasis [17]. Timely anti-fungal therapy and controlling the source of the infection are crucial de-terminants in terms of the survival of patients that have been infected by invasive candidiasis [18].

Glucan, which is a major component of the cell walls of many fungi, with either 1,3-P-linkages or 1,6-P-linkages, is also one of the main structural components forming the cell wall of *C. albicans* [19], and this species produces glucanases. *C. albicans* can thus be used as a model system for analyzing the properties of certain cell-wall-degrading enzymes, and can also be used to study the role of such enzymes in growth and morphogenesis [20]. It has been observed that the specificity and sensitivity of serum BDG testing for the diagnosis of invasive candidiasis have been seen to range from 56% to 93% and 57% to 97%, respectively. In a recent meta-analysis comprised of 11 studies, the sensitivity was around 75% [21]. Thus, developing a method for the detection of BDG can complement blood culture analysis and can improve the diagnostic capabilities of the current testing procedures.

Hemicellulase can act on glucan and hydrolyze it to produce glucose, which can be used to break down the cell walls of fungi such as *C. albicans*. This mechanism can be tracked by measuring the levels of glucose produced in the medium over time, and can also act as an identifier for the presence of fungi, such as *C. albicans,* in the system.

Diabetic patients need to keep monitoring their blood glucose levels regularly; thus, the use of personal glucose meters is becoming more common [22]. Mostly, the personal glucose meter measures glucose levels in the bloodstream using amperometric methods. Amperometric methods constitute measuring the current produced by the electrons released during the oxidation of D-glucose to D-gluconolactone. [23]. In the case of glucose monitoring, the glucose taken up in the blood sample is made to react with an enzyme electrode that contains glucose oxidase (or dehydrogenase). The process then involves re-oxidation of a series of compounds such as the enzyme by a mediator reagent in excess, such as a ferrocene derivative, ferricyanide ion, or osmium bipyridyl complex, which in turn is re-oxidized by the reaction taking place at the electrode, which generates an electric current. The total charge in the electrode that is required as a result of the re-oxidation process is proportional to the amount of glucose in the blood sample that has been through the reaction with the enzyme [24].

Thus, it is possible to use enzymes such as hemicellulase to break down the cell walls of fungi, like *C. albicans,* that might be present in the system, and use personal glucose meters to measure the amount of glucose that is produced as a result of the breakdown of glycan in the cell walls of the fungi in order to predict the amount of fungi that might be present. To develop a system that can detect the amount of fungi, such as *C. albicans*, in a system, some technical barriers must be overcome. These include figuring out the amount of enzymes required for accurate detection, accounting for the influence of other factors such as the presence of external glucose in the sample taken, and the presence of Gram-positive and Gram-negative bacteria, which that might influence the reading produced.

Certain techniques can be employed for rapid candida detection, which also offer the possibility of integrating ELISA. Techniques such as the use of micromechanical cantilever arrays that can be functionalized and coated for the selective identification of certain fungi have been developed [25], which can be adapted for the detection of *C. Albicans*. Another technique for the rapid detection of fungi that can be adapted to the detection of candida involves the use of label-free biosensors based on field effect transistors (FET) and single-wall carbon nanotubes [26]. A non-culture-based method that uses a lateral flow immunoassay for the detection of invasive candidiasis has also been developed [27]. The use of a polymerase spiral reaction for the detection of *C. Albicans* can also be employed, which can offer effective results within a short period, while maintaining accuracy [28]. Electrochemical sensors can also be used for the detection of candida that can be functionalized with anti-candida antibodies for specificity, which detects the presence of candida using electrochemical impedance spectroscopy and offers fairly accurate and fast results [29]. A probe-based design that involves the usage of nanoporous anodic alumina and specific oligonucleotides that can specifically detect the DNA of *C. Albicans* and can thus be used for rapid candida detection, has also been developed and shows a high sensitivity and specificity for detection [30]. Therefore, we see that that most work is being focused on techniques and methods that can rapidly and easily detect candida non-invasively, and that can offer a high sensitivity and accuracy to match the current gold standard in the field, which is culturing cells taken from blood samples. Other novel biosensing techniques, e.g., Brownian nanobeads [31,32,33,34,35], surface plasmon resonance [36], liquid crystal [37,38], nano-gold clusters [39], and flow cytometers [40,41,42], can also be the potential techniques for Candida detection.

**Figure 1:**
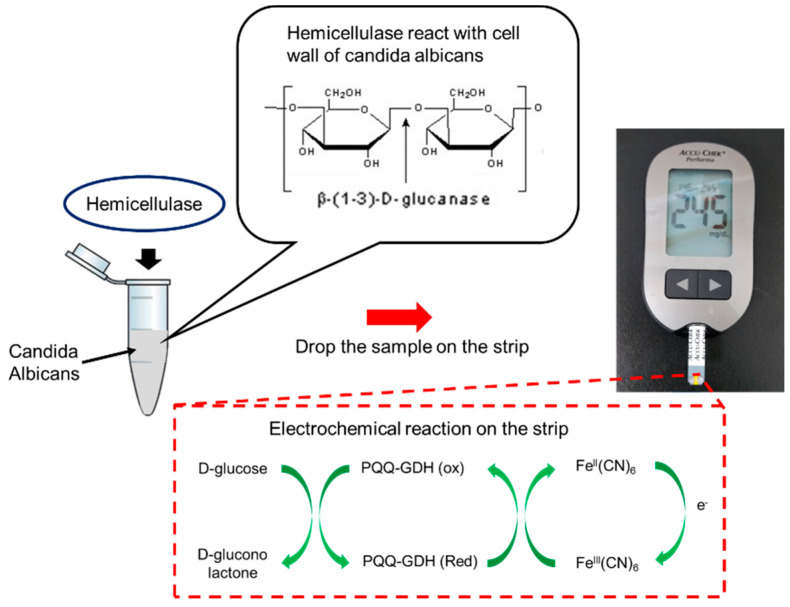
Mechanism of Candida detection. When adding hemicellulase into the sample, the cell walls of *C. albicans* will be broken down into oligo and glucose, which can be detected by a commercial glucose meter. PQQ—pyrroloquinoline quinone; GDH—glucose dehydrogenase; FeIII(CN)_6_—potassium hexacyanoferrate (III); FeII(CN)_6_—potassium hexacyanoferrate (II).

In our previous study, we used electrochemical sensors to monitor endogenous β-galactosidase of *E. coli* for sensing bacteria [43]. In this study, we looked to estimate the level of Candida infection present in the system using personal glucose meters as the measuring device in order to act as early detection systems to increase the efficiency of the current diagnostic procedure and to help the prevention of Candidiasis. The Accu-chek Performa (F. Hoffmann-La Roche AG, Basel, Switzerland) glucose meter was used in the experiment for all glucometric measurements, which can detect glucose levels in the range of 0.6–33.3 mmol/L or 10–600 mg/dL, with a sensitivity of about 1 mmol/L. Furthermore, any glucose meter that has a range and sensitivity comparable to that of the Accu-chek Performa can be used for testing [44]. By using a personal glucose meter, the detection was instant and reliable within a very small volume of samples (5 µL). The detection system and strips were also cheap. This method can rapidly screen yeast or bacteria for further clinical treatment. The sensing mechanism and procedure are shown in Figure 1. The cell walls of Candida can break down when adding hemicellulase into the samples. After reacting for a certain time, the samples were dropped onto a personal glucose meter to measure the glucose level. Therefore, the level of Candida infection could be estimated by comparing the glucose levels to those before adding hemicellulase.

## 2. Materials and Methods

### 2.1. Candida Cell Culture

*C. albicans* were obtained from Dr. Chung-Yu Lan’s Lab (Institute of Molecular and Cellular Biology, National Tsing Hwa University, Hsinchu, Taiwan) and were grown in a yeast-extract peptone dextrose (YPD) medium (1% yeast extract, 2% peptone, and 2% glucose) or synthetic complete (SC) medium (0.67% yeast nitrogen base with ammonium sulfate, 2% glucose, 0.079% complete supplement mixture of amino acids; MP Biochemicals, Solon, OH, USA). All of the reagents were purchased from Sigma-Aldrich (St. Louis, MO, USA), unless otherwise indicated. To culture *C. albicans* to make it the pseudohypha type, an RPMI 1640 medium was used. The *C. albicans* were suspended in 5 mL of the RPMI medium and shaken at 100 RPM for 24 h. Glycan was also obtained from Sigma, and was used in the testing process to evaluate the glucose meter. Hemicellulase was used as a cell wall degrading enzyme, and was purchased from Sigma Aldrich. Urine; the serum and blood samples were obtained from healthy donors. This protocol was approved by the Institutional Review Board of Taipei Veterans General Hospital (IRB 2019-01-021CC approved on 22 January 2019).

### 2.2. Sample Measurement

The aim of the testing was to find out how much enzymes were required to produce a discernible reading for glucose levels measured using a glucose meter. When the hemicellulase was added to the *C. albicans* sample or to a solution containing glucan, which is a major constituent of the cell walls of *C. albicans*, the glucan are broken down into glucose and oligo. Further testing involved finding out whether the presence of certain external influences, such as an initial level of glucose in the system or the presence of Gram-positive or Gram-negative bacteria, could influence the glucose meter reading measured during this analysis. First, hemicellulase was added to the *C. albicans* sample to find out if the cell wall degrading the activity of the enzyme was enough to produce a reading on the glucose meter. Then, to find out the optimal concentration and amount of hemicellulase used for the detection process, the cell wall degrading activity of hemicellulase on *C. albicans* was analyzed. *C. albicans* were also spiked in different body fluids, including urine and serum, in order to mimic the detecting situation.

## 3. Results

### 3.1. Glucan Sensing for System Optimization

**Figure 2:**
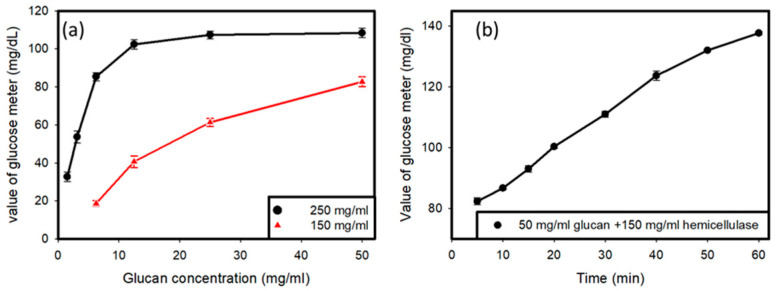
(**a**) Reading of the glucose meter versus the glucan concentration tested with different values of hemicellulase. (**b**) Reading of glucose meter over time with fixed values of glucan (50 mg/mL) and hemicellulase (150 mg/mL)**.**

Two samples were prepared, one with a concentration of hemicellulase of 250 mg/mL and another with the concentration of hemicellulase set at 150 mg/mL (Figure 2a). The amount of glucan was varied, and different amounts of glucan were tested with the two hemicellulase concentrations in order to obtain a curve for the variations in the glucose reading with variations in the amount of glucan with a fixed concentration of hemicellulase as the enzyme in the solution. It was determined that hemicellulase with a concentration of 150 mg/mL was appropriate for further usage, and all further tests involved the use of hemicellulase at a concentration of 150 mg/mL. Then, the value produced by the glucose meter was tested when 50 mg/mL of glucan was added to a solution containing 150 mg/mL of hemicellulase over time, in order to observe the variations that occurred in the glucose reading over time when fixed concentrations of hemicellulase and glucan were used. The tests were carried out for 1 h, with readings taken at intervals every 10 min.

Preliminary testing, as shown in Figure 2a, was done to find out the optimal concentration of hemicellulase to be used for the experiment, and it showed that when 250 mg/mL of hemicellulase was added to the solution containing *C. Albicans*, there was a saturation of glucose produced by the reaction upon the addition of glucan to the solution. Therefore, a lower concentration of 150 mg/mL of hemicellulase was used. It was observed that saturation did not occur in the glucose readings. Another testing of the solution containing 50 mg/mL of glucan and 150 mg/mL of hemicellulase over time showed an increase in the glucose reading on the meter, as shown in Figure 2b. It indicated that the cell wall degradation activity of the enzyme was a time-consuming process. As more and more glucan hydrolyzed over time, higher amounts of glucose were produced in the solution.

### 3.2. C. albicans Detection

*C. albicans* was cultured in an RPMI medium against an increase in hemicellulase concentration in order to assess the variations in the glucose levels when *C. albicans* present in a culture medium was subjected to an increase in hemicellulase concentration. The cell culture was prepared using Candida cells grown in an RPMI medium over a period of 24 h. Testing was done over a period of 24 h at 37 °C, with the solutions being shaken at 100 RPM for preparation. The cell-filtered RPMI medium after culturing *C. albicans* for 24 h was used as the control to establish the difference if any existed.

**Figure 3:**
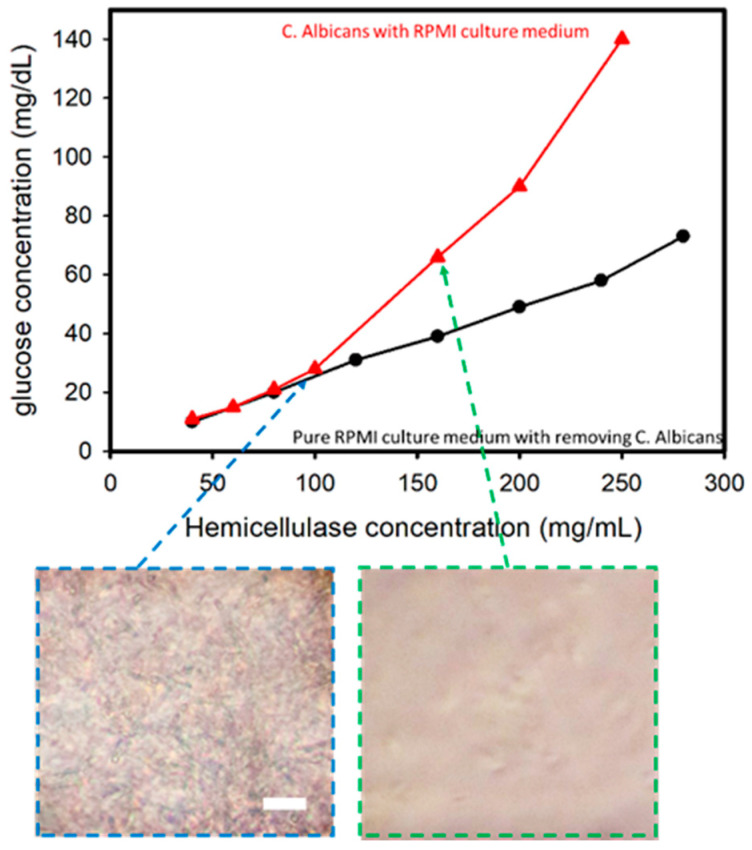
Glucose concentration versus hemicellulaose concentration for *Candida albicans* in a RPMI culture medium. The inserted images show that when concentration of hemicellulase is higher than 150 mg/mL, *C. albicans* can be lysed in 1 min (incubation time of 1 min). Scale bar: 50 μm.

In this experiment performed using *C. albicans* cultured in an RPMI medium, it was found that with the increase in hemicellulase concentration, higher amounts of cell wall degradation activity occurred, thus leading to a higher concentration of glucose in the solution. The comparison of samples with and without removing *C. albicans* is shown in Figure 3. There was a sharp increase in the glucometric readings for the solution containing *C. albicans* compared with the solution without one when the hemicellulase concentration was higher than 100 mg/mL. The sub-images reveal the optical microscopic images after mixing the hemicellulase for 1 min. When the hemicellulase concentration was 160 mg/mL, the *C. albicans* were analyzed and compared to the sample with a hemicellulase concentration of 100 mg/mL. This indicated that the presence of *C. albicans* was the reason for the increase in glucose in the medium.

### 3.3. Spiked Sample Test

**Figure 4:**
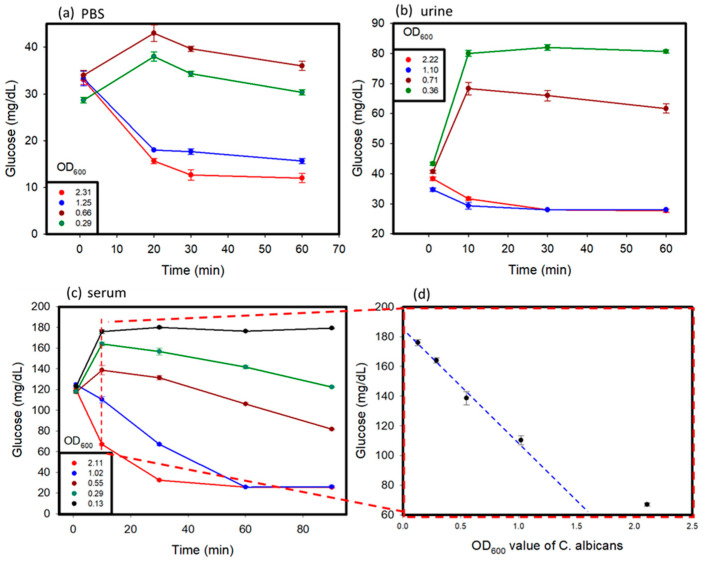
Spike sample test: *C. albicans* was suspended in (**a**) PBS, (**b**) urine, (**c**) serum solution, and (**d**) serially diluted samples to prepare spiked samples with varying concentrations. The OD_600_ values of the samples were measured before testing.

*C. albicans* affects different parts of the body, and glucose values produced by the *C. albicans* solution might vary in different conditions [17]. In this study, *C. albicans* was added to different solutions, including PBS, human serum, and human urine, to study its detection by using a personal glucose meter. The testing of these samples was carried out over a time period of 1 to 2 h. The results are shown in Figure 4a–c. Various concentrations of *C. albicans* in different solutions were used to discern the differences in glucose concentrations that occurred over time, with a hemicellulase concentration of 150 mg/mL being used. It was observed that at a lower concentration of *C. albicans* in the sample, the glucose concentrations rose over time because of the breakdown of their cell walls, as observed in previous tests. When the reaction time was over 10 min, the glucose levels began to decrease, because the endogenous enzymes were released from the cells and dissolved the glucose in the medium. With higher concentrations of *C. albicans*, due to large amount of endogenous enzymes released, the glucose produced was consumed very fast, which lead to a drop of glucose levels in the samples over time. The glucose levels in the urine and PBS reached their threshold values faster than those observed in the blood serum. This can be attributed to the presence of external glucose in the blood serum, which provided additional glucose for consumption.

In summary, in the beginning, because the activity of endogenous enzymes was low when the OD_600_ values of *C. albicans* were lower than 1, the glucose values increased. After 10 min, the ability of the cell walls that were broken down by hemicellulase became weak, and the endogenous enzymes were released from *C. albicans* to dissolve the glucose. The glucose values kept decreasing. When the OD_600_ values of *C. albicans* were greater than 1, the glucose dissolution rate of the endogenous enzymes was higher than the cell wall break down rate. We think that the cells might have released too many endogenous enzymes, keeping the glucose dissolution ability high.

Furthermore, for the *C. albicans* detection in the serum, we looked at the results of measuring at 10 min and the plot of the OD_600_ values of *C. albicans* versus glucose concentration in Figure 4d. The results revealed that glucose concentrations versus OD_600_ values showed a linear decrease when the OD_600_ values of *C. albicans* were lower than 1. This could become a detection protocol. The 1 mL serum samples after adding 150 mg/mL hemicellulase were incubated for 10 min. We measured the glucose levels and compared them to the results obtained without adding hemicellulase. The concentration of *C. albicans* could be estimated based on Figure 4d. When the concentration of *C. albicans* was lower than 0.13 OD_600_, *C. albicans* could not be detected.

**Figure 5:**
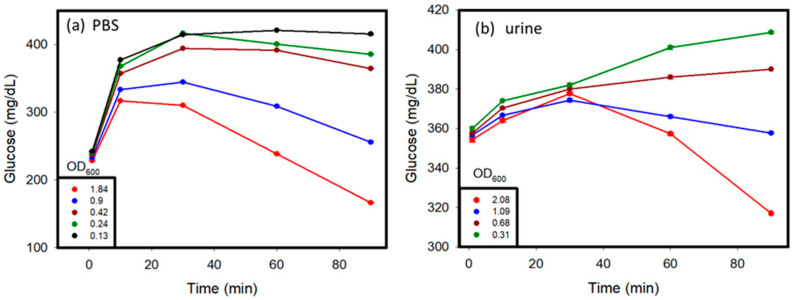
Glucose added to urine before testing, showing (**a**) glucometric responses with different OD_600_ values taken before testing *C. albicans* over time in PBS and (**b**) glucometric responses with different OD_600_ values taken before testing *C. albicans* in urine, when glucose was initially added to the sample before testing.

Glucose was initially added to the PBS and urine samples, and then the samples were tested to observe the change in glucose values over time so as to ascertain the difference between the samples that did not contain glucose initially (Figure 5). The presence of glucose in the system where the initial glucose level of the system was higher than that in the case of PBS and urine samples without glucose, is indicated by the glucometric readings in Figure 4a,b. Over time, samples with a higher concentration of *C. albicans* showed an initial increase in glucose levels because of the cell wall dissolution, and then a decrease in the glucose levels because of the consumption of glucose by the released endogenous enzymes. This trend was proven by the fact that at very low concentrations of *C. albicans*, less endogenous enzymes from *C. albicans* consumed glucose in samples. An increase in glucose levels as a result of cell wall dissolution was observed. In contrast, at higher concentrations of *C. albicans*, more endogenous enzymes were released from the cells after lysing, and were able to rapidly consume glucose in the media, and therefore exhibited an apparent decrease in the glucose levels after hitting a maximum value. Similar trends were observed in both the PBS and urine samples.

## 4. Conclusions

The current gold standard for *C. albicans* diagnosis still remains the cell culture process, taken from the affected region or from a blood sample. These methods take a long time to produce results, as a result of which patients might suffer. Thus, it is necessary to come up with early and fast detection systems that can assist in the diagnostic process. In this study, we demonstrated that the measurement of change in glucose levels during the degradation of the cell walls of commensal fungi, such as *C. albicans*, is an effective method for predicting and ascertaining the presence of such fungi in the system. A difference in the initial presence of glucose in the system resulted in a difference in the glucometric response. Appropriate times for testing can produce viable results. The glucometric response over time has been ascertained to be a function of the initial concentration of the glucose in the system, as well as the amount of hemicellulase used for testing. The developed mechanism for *C. albicans* detection using the electrochemical sensing technique can act as an early indicator of invasive candidiasis in order to help prevent the spread of the infection and save the patient time and money in treatment.
